# Supplementary material for: Exploring tumor-normal cross-talk with TranNet: Role of the environment in tumor progression
Source: PLoS Comput Biol. 2023 Sep 18;19(9):e1011472. doi: 10.1371/journal.pcbi.1011472 (PMC10538798; doi:10.1371/journal.pcbi.1011472)
Supplement: S1 Text — Tables A and B, Figs A and B and C and D and E and F and G. (PDF) [file pcbi.1011472.s001.pdf]

Supplemental Information

Supplementary Table

KEGG pathways	LUAD (PC3)		PRAD (PC3)	
	p-value	q-value	p-value	q-value
Cell cycle	3.3E-15	4.3E-14		
DNA replication	6.6E-12	4.3E-11		
Pyrimidine metabolism	1.4E-08	6.3E-08		
Spliceosome	1.4E-05	4.8E-05	4.4E-07	8.8E-06
Oocyte meiosis	3.2E-05	8.5E-05		
Nucleotide excision repair	4.3E-05	9.2E-05	0.00176	0.0035
Purine metabolism	4.9E-05	9.2E-05		
Ether lipid metabolism			2.1E-05	0.00021
Mismatch repair	0.00014	0.00021		
Homologous recombination			0.00014	0.00021
Base excision repair	0.00029	0.00038		
Pathways in cancer	0.00131	0.00154	0.00254	0.00438
Vibrio cholerae infection			0.00046	0.00202
Mapk signaling pathway			0.00046	0.00202
Aminoacyl trna biosynthesis			0.00065	0.00202
Proximal tubule bicarbonate reclamation			0.00065	0.00202
Endocytosis			0.00070	0.00202
Adherens junction			0.00089	0.00223
Glycerophospholipid metabolism			0.00126	0.00280
Neuroactive ligand receptor interaction			0.00263	0.00438
Progesterone mediated oocyte maturation	0.00442	0.00479		
Basal cell carcinoma			0.00412	0.00625
Inositol phosphate metabolism			0.00464	0.00625
Calcium signaling pathway			0.00469	0.00625
Arginine and proline metabolism			0.00642	0.00802
VEGF signaling pathway			0.00780	0.00918

Table A. Functional enrichment for principal components. Kegg pathways enriched for targets of differentially expressed principal components selected in the list of the high scoring predictors.

		BRCA	LUAD	LUSC	PRAD	LIHC
Number of patients		105<br>Normal-like: 2;<br>Her2: 9;<br>Basal: 14;<br>Luminal-B: 20;<br>Luminal-A: 53;<br>Not-reported: 7;	57	50	47	40
Age at diagnose	$20 \leq$ age < 50	36	4	1	4	7
	$50 \leq$ age ≤ 70	49	32	29	40	16
	$70 <$ age ≤ 90	20	21	20	3	17
Gender	Male	1	24	37	47	21
	Female	104	33	13	0	19
Ethnicity	Not hispanic or latino	81	43	30	45	38
	Not reported	24	14	20	2	2
Race	White	97	53	41	39	30
	Black or african american	6	4	4	7	6
	Asian	1	0	0	0	3
	Not reported	1	0	5	1	1

Table B. Clinical cohorts. Clinical cohorts of the patients' whose matched normal and tumor samples are available in TCGA data portal. Age at diagnose, gender, ethnicity and race are summarized. Expression based subtype information for BRCA patients are obtained from a previous study https://www.nature.com/articles/nature11412.

Supplementary Figures

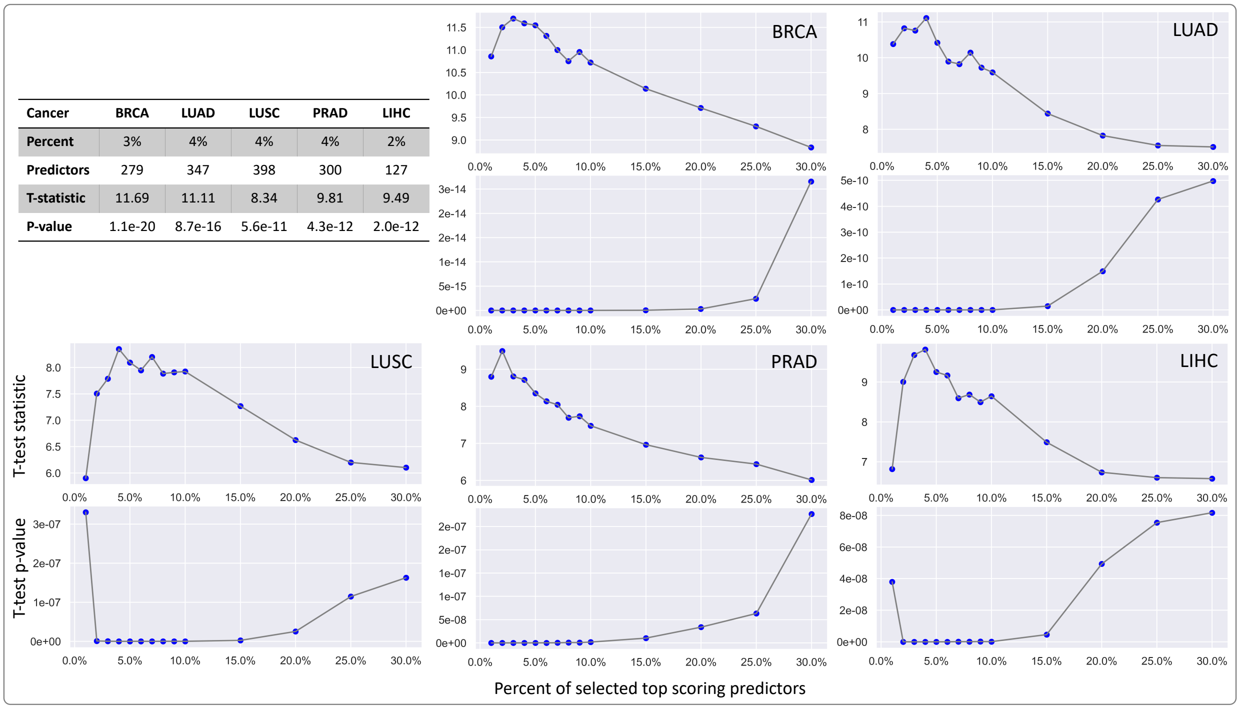

Cancer	BRCA	LUAD	LUSC	PRAD	LIHC
Percent	3%	4%	4%	4%	2%
Predictors	279	347	398	300	127
T-statistic	11.69	11.11	8.34	9.81	9.49
P-value	1.1e-20	8.7e-16	5.6e-11	4.3e-12	2.0e-12

Fig A. Selection of the top predictors. Paired-sample T-test was used for evaluating prediction power of TranNet. Depending on the number of the top PP scoring predictors, the prediction accuracy varies for each of the five cancers as visualized in the figures. The cut-off number describing the predictor set is decided based on the percents varying from 1% to 30%. For each of the selected sets of predictors, the leave-one-out test was performed over patients. As an illustrative example, for predicting the tumor expression $y_{i(\cdot)}$ of a patient i (105 patients for BRCA), the transition matrix $M_{(-i)}$ of size 279×9309 is inferred from the expressions of the top scoring 279 predictors in normal and the 9308 genes and 1 principal component in tumor (for 104 patients except for patient i). Then $M_{(-i)}$ is used as the transition mapping to predict the tumor tissue expression $y_{i(\cdot)} \in R^{9309}$ from the given normal expression $x_{i(\cdot)} \in R^{9309}$.

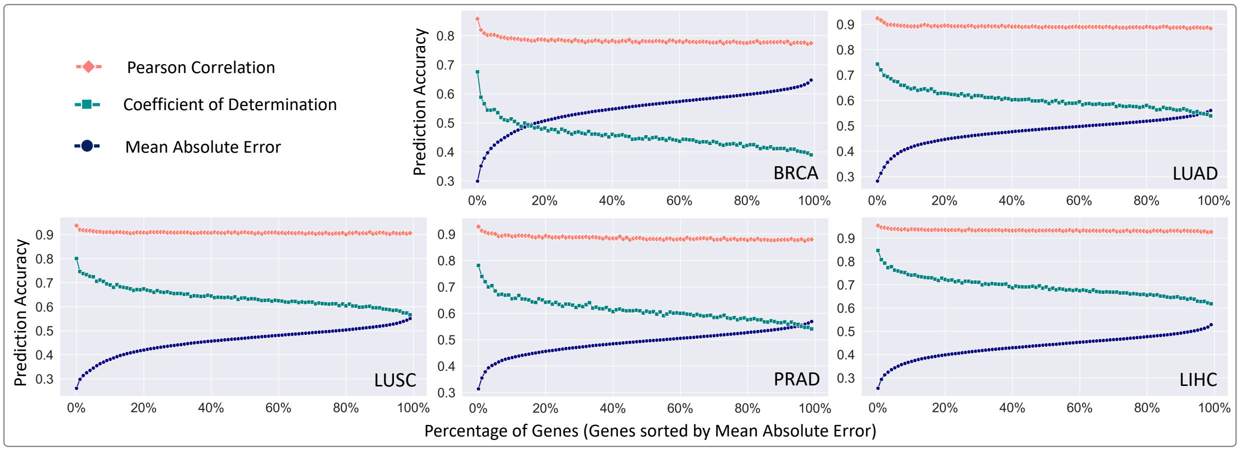

Fig B. The gene-wise prediction accuracy in three different measurements between the predicted and the original expressions. The horizontal axis denotes genes sorted by their prediction error (mean absolute error) while the prediction accuracy is represented in the vertical axis. For each cancer type, the sorted list of the target genes is divided into 100 intervals including the same number of genes, and the prediction accuracy in each interval is represented by the average error in the interval.

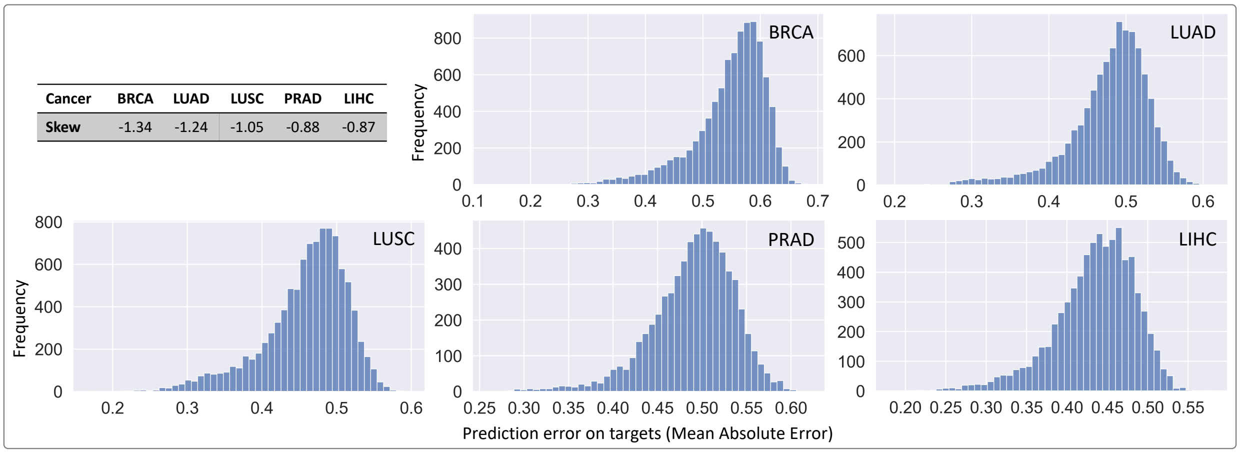

Cancer	BRCA	LUAD	LUSC	PRAD	LIHC
Skew	-1.34	-1.24	-1.05	-0.88	-0.87

Fig C. Distributions of the approximation errors on the target genes. The horizontal axis denotes the mean absolute error between the original expression and the predicted expression for a gene. In other words, for each gene, its prediction error is the absolute summation over sample-wise errors. Vertical axis denotes the corresponding frequency for the gene-wise prediction accuracy.

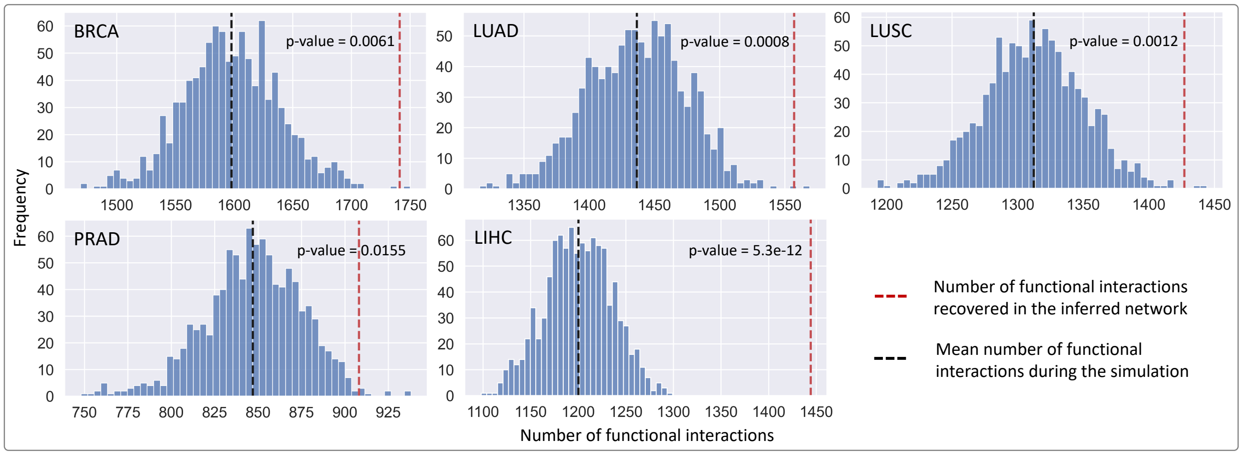

Fig D. Enrichment of the TranNet networks in functional interactions. Horizontal axis represents the number of functional interactions recovered in randomized networks whereas vertical axis shows the corresponding frequencies. Doted-red line corresponds to the TranNet network. The p-value of the enrichment in functional interactions in the TranNet network relative to random and computed based on z-score test.

Cancer	BRCA	LUAD	LUSC	PRAD	LIHC
Correlation	0.96	0.96	0.95	0.94	0.95
Average degree	32.3	27.1	25.8	25.1	22.5

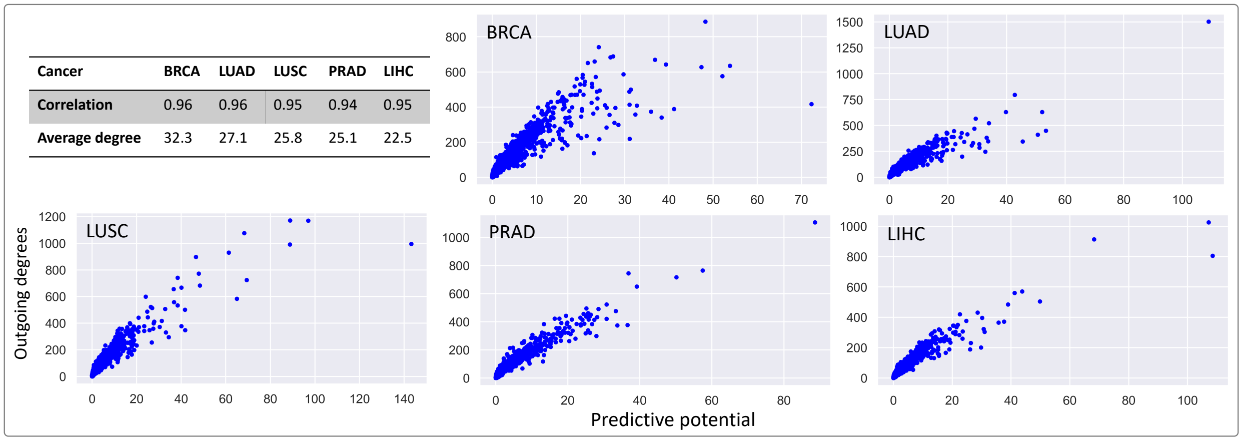

Fig E. Joint distribution of PP scores and outgoing degrees. Scatter plots visualize the joint distribution of predictive potentials (PP) (horizontal axis) and outgoing degrees (vertical axis) of the genes and principal components with nonzero predictive potentials. Dotted-red line denotes the predictive potential cut-off for the selected high PP scoring predictors. Correlation: Pearson correlation between predictive potentials and outgoing degrees of the genes. Average degree: average outgoing degree of the genes and principal components having nonzero predictive potentials in the network. Average PP: average predictive potential of the genes and principal components having nonzero predictive potentials in the network.

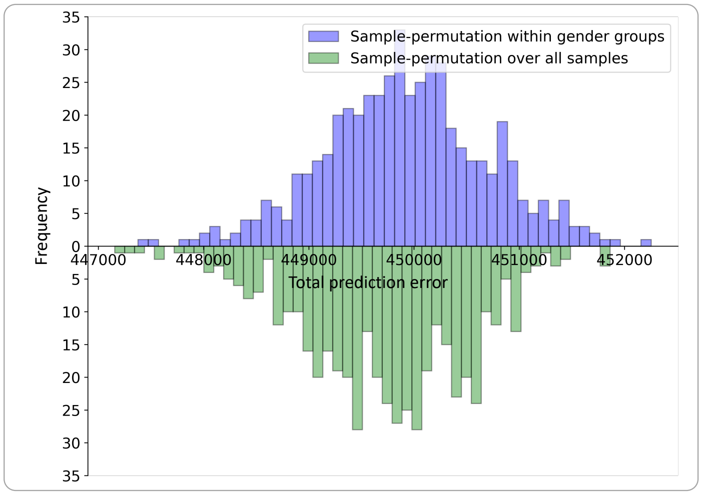

Fig F. Distribution of the total prediction error. The histograms visualize the distributions of the total prediction errors generated from two different settings of permutations. The samples are separately permuted within each gender group (purple). The same permutation test was performed without controlling for gender (green).

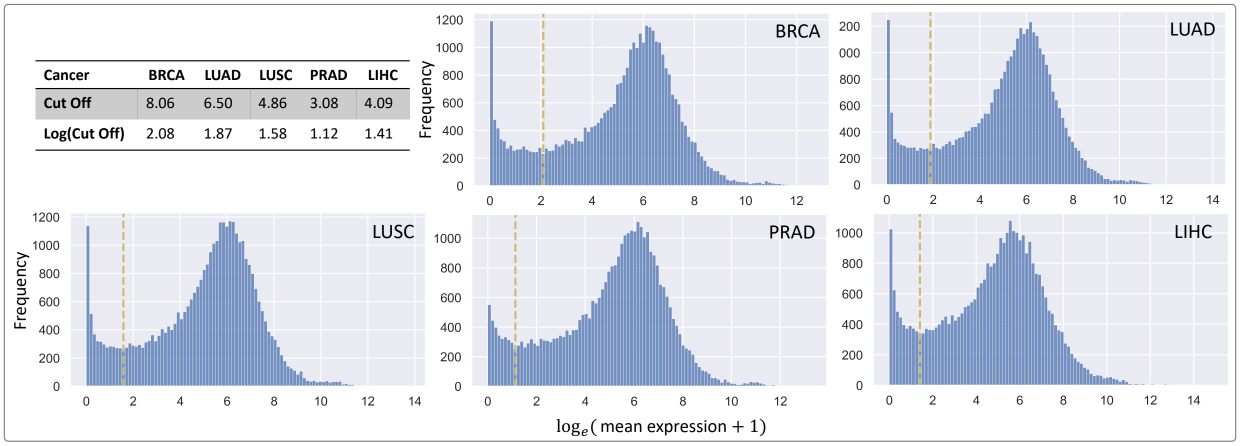

Cancer	BRCA	LUAD	LUSC	PRAD	LIHC
Cut Off	8.06	6.50	4.86	3.08	4.09
Log(Cut Off)	2.08	1.87	1.58	1.12	1.41

Fig G. Distribution of expression level. The histograms visualize the distributions of log_e(mean expression + 1) for the five cancers. For each cancer, the expression level cut-off was described by the smallest frequency in the range between two peaks in the distribution.
